# The Blood Immune Cell Count, Immunoglobulin, Inflammatory Factor, and Milk Trace Element in Transition Cows and Calves Were Altered by Increasing the Dietary n-3 or n-6 Polyunsaturated Fatty Acid Levels

**DOI:** 10.3389/fimmu.2022.897660

**Published:** 2022-07-07

**Authors:** Xiaoge Sun, Yuhuang Hou, Yue Wang, Cheng Guo, Qianqian Wang, Yan Zhang, Zhantao Yang, Zhonghan Wang, Zhijun Cao, Wei Wang, Shengli Li

**Affiliations:** ^1^ State Key Laboratory of Animal Nutrition, Beijing Engineering Technology Research Center of Raw Milk Quality and Safety Control, College of Animal Science and Technology, China Agricultural University, Beijing, China; ^2^ Laboratory for Animal Production and Animal Product Quality, Department of Animal Sciences and Aquatic Ecology, Faculty of Bioscience Engineering, Ghent University, Ghent, Belgium; ^3^ Animal Production Systems group, Wageningen University & Research, Wageningen, Netherlands; ^4^ School of Agriculture, Ningxia University, Yinchuan, China

**Keywords:** dairy cows, neonatal calves, immune cell, inflammation, colostrum

## Abstract

Transition dairy cows experience sudden changes in both metabolic and immune functions, which lead to many diseases in postpartum cows. Therefore, it is crucial to monitor and guarantee the nutritional and healthy status of transition cows. The objective of this study was to determine the effect of diet enriched in n-3 or n-6 polyunsaturated fatty acid (PUFA) on colostrum composition and blood immune index of multiparous Holstein cows and neonatal calves during the transition period. Forty-five multiparous Holstein dairy cows at 240 days of pregnancy were randomly assigned to receive 1 of 3 isoenergetic and isoprotein diets: 1) CON, hydrogenated fatty acid (control), 1% of hydrogenated fatty acid [diet dry matter (DM) basis] during prepartum and postpartum, respectively; 2) HN3, 3.5% of extruding flaxseed (diet DM basis, n-3 PUFA source); 3) HN6, 8% of extruding soybeans (diet DM basis, C18:2n-6 PUFA source). Diets containing n-3 and n-6 PUFA sources decreased colostrum immunoglobulin G (IgG) concentration but did not significantly change the colostrum IgG yield compared with those with CON. The commercial milk yield (from 14 to 28 days after calving) was higher in the HN3 and HN6 than that in the CON. Furthermore, the n-3 PUFA source increased neutrophil cell counts in blood during the prepartum period and increased neutrophil percentage during the postpartum period when compared with those with control treatment. Diets containing supplemental n-3 PUFA decreased the serum concentration of interleukin (IL)-1β in maternal cows compared with those in control and n-6 PUFA during prepartum and postpartum. In addition, the neonatal calf serum concentration of tumor necrosis factor (TNF) was decreased in HN3 compared with that in the HN6 treatment. The diet with the n-3 PUFA source could potentially increase the capacity of neutrophils to defend against pathogens in maternal cows by increasing the neutrophil numbers and percentage during the transition period. Meanwhile, the diet with n-3 PUFA source could decrease the pro-inflammatary cytokine IL-1β of maternal cows during the transition period and decline the content of pro-inflammatary cytokine TNF of neonatal calves. It suggested that the highest milk production in n-3 PUFA treatment may partially be due to these beneficial alterations.

**Graphical Abstract:**
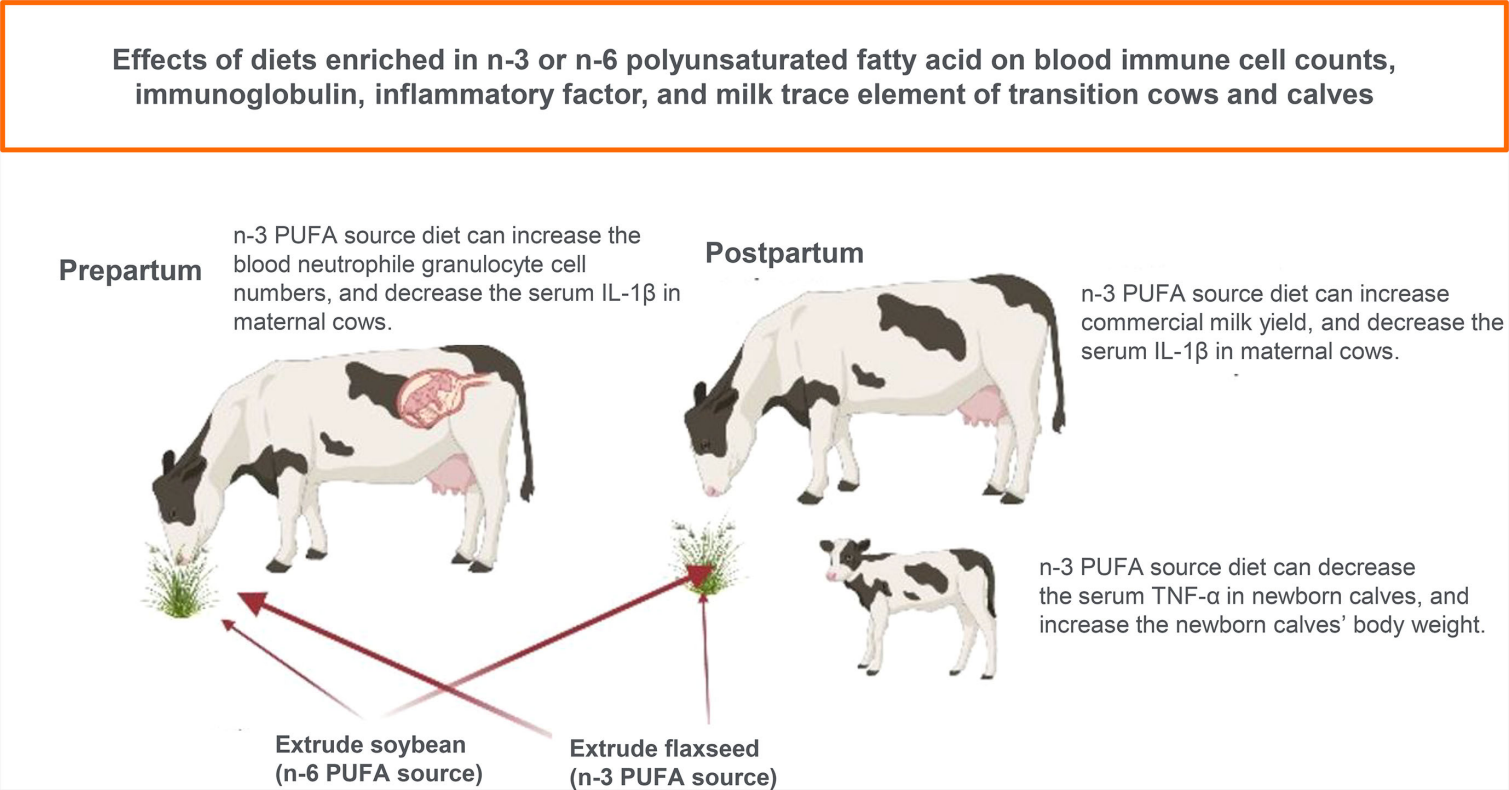
Effects of diets enriched in n-3 or n-6 polyunsaturated fatty acid on blood immune cell counts, immunoglobulin, inflammatory factor, and milk trace element of transition cows and calves.

## Introduction

The transition period of dairy cows, which normally spans from 3 weeks before parturition and 3 weeks after, is characterized by dramatic changes in metabolism and host defense system that are associated with the increased incidence of diseases such as retained placenta, milk fever, ketosis, and clinical mastitis (1, 2). Furthermore, it is suggested that metabolic adaptations such as lipid mobilization are accompanied by alterations in inflammatory responses that modify the immune function (3). Bertoni et al. (4) revealed that the cows with the strongest inflammatory profiles were at an 8-fold higher risk for experiencing one or more transition disorders, which led to less milk production in the period of first-month lactation.

Many types of evidence showed that the immune system was impared during the transition period, including decreased mitogen-induced proliferation of lymphocytes, decreased antibody response, and decreased capacity of neutrophils to kill pathogens (5). One strategy to improve the metabolic status of transition cows is supplementing fatty acids (FAs), which can increase dietary energy density (6, 7) and modulate the immune cell function and inflammatory response (8). Fatty acids can modify the immune response in several pathways, which include the inhibition of arachidonic acid metabolism, induction of anti-inflammatory mediators, modification of intracellular lipids, and activation of nuclear receptors (9, 10). Studies in cultured cells, animal models, and human subjects have shown that both the dose and type of FA can influence the immune response (11). In addition, it has been shown that a colostrum supplement of n-3 FA can reduce the oxidant status of newborn calves in the first week of life (12) and encourage a greater anti-inflammatory state (13).

However, most of the meta-analyses regarding the effects of different types of lipid supplements on dairy cows focused on the milk FA and productivity (14), rarely involved in the blood immune cell counts, inflammatory cytokine levels in maternal cows, and whether these impacts in pre-calving maternal cows will directly influence the newborn calves. Moreover, most of the colostrum studies focus narrowly on immunoglobulin G (IgG), IgA, and IgM and ignore other nutrients or compounds (15). A complete understanding of transition cow biology requires a truly integrative perspective. Therefore, it is necessary to determine changes of immune cells, inflammatory cytokines in maternal cows and neonatal calves, and the effect of diet supplementary n-3 or n-6 polyunsaturated fatty acid (PUFA) on immune function and inflammatory reaction in cows during the transition period. We hypothesize that n-3 PUFA supplementation in the diet can improve cows’ transfer from non-lactation to lactation initial during the perinatal period.

In this study, multiparous dairy cows were fed diets enriched in saturated FA (C16:0), extruded flaxseed (n-3 PUFA source), or extruded soybean (n-6 PUFA) during the transition period. The objective of this study is to assess the influence of dietary PUFA and parturition on milk production, colostrum composition, blood immune cell counts, immunoglobulin, and inflammatory cytokines in transition cows and calves.

## Materials and Methods

Animals involved in this experiment were taken care of according to the guidelines from the committee of animal welfare and animal experimental ethical inspection of China Agricultural University. The committee reviewed and approved the experiment and all procedures involving animals (protocol number: CAU20201024-2).

### Animals and Experimental Design

Multiparous Holstein dairy cows (n = 45), at 240 days of pregnancy, were randomly assigned to 3 isoenergetic and isoprotein treatments: 1) 1% dry matter (DM) hydrogenated FA (C16:00 enriched) in the diet from 28 days prepartum to 28 days postpartum (CON); 2) 3.5% DM of extruded flaxseed (C18:3n-3 enriched) supplemented in the diet from 28 days prepartum to 28 days postpartum (HN3); 3) 8% DM of extruded soybean (C18:2n-6 enriched) supplemented in the diet from 28 days prepartum to 28 days postpartum (HN6). The cows were dried at 215 days of pregnancy (the duration of dry period was 60 ± 3 days). Holstein dairy cows were housed in a free-stall barn (with rubber bed and rice hull bedding) with the Roughage Intake Control (RIC) system (INSENTEC, Marknesse, Netherlands) and offered a total mixed ration (TMR) twice daily at 07:00 and 15:00 h *ad libitum* prepartum and postpartum. The ingredients and chemical composition of the close-up and milking cow TMR were presented in **Table S1**. The calves were separated from their mother immediately after being born. They were placed in individual hutches deep-bedded with rice hull, weighed, blood sampled, and given 4 L of colostrum pooled from their mother. The procedure was completed generally within 2 h after the calf was born. The colostrum feeding was repeated at 12 h and again at 24 h, and the blood was collected again at 24 h. After 24 h, the calves were fed whole milk until weaning at 55 ± 3 days of age. The animal feeding experiment was carried out in Beijing Zhongdi Animal Husbandry Technology Co. Ltd. (39°30’N, 116°33’E) in northern China. The average temperature, humidity, altitude, and light/dark cycle in the farm were 1°C–12°C, 58%, 35 m, and 16/8 h, respectively.

Of the total 45 cows assigned to treatments, only the data of 37 of them were used in the trial at the end (12 cows in the CON group, 12 in the HN3 group, and 13 cows in the HN6 group). Two cows that calved early and six diseased cows were excluded in the statistical analysis (**Table S2**). The parity, body weight (BW), body condition score (BCS), and milk yield of the cows were 2.47 ± 1.06, 759.23 ± 65.89 kg, 3.30 ± 0.26, and 10,182.18 ± 1,664.90 kg/lactation (mean ± SD, previous lactation production) at the start of the experiment, respectively, and they were similar in the three groups (**Table S3**). The detailed health information of the calves was shown in **Table S4**.

### Measurements

Samples of TMR and orts were obtained weekly and dried at 55°C for 48 h for DM determination. The TMR samples were pooled weekly and sent to the State of Key Laboratory of Animal Nutrition (China Agricultural University, Beijing) for chemical composition analysis. Postpartum cows were milked four times daily at 06:00, 12:00, 18:00, and 24:00 h, and the milk production was recorded at each milking by the ALPROTM system (DeLaval^©^, Tumba, Sweden). The colostrum was collected using a Portable Milking Machine (H8192, Duomai Technology Co., Ltd., Hebei, China) immediately after the cow calving and then two 50-ml aliquots of colostrum were obtained for further analysis. Two 50-ml aliquots of milk were collected at 14 and 28 days after calving and at each milking proportional to yield (4:3:3, composite). One aliquot of milk or colostrum containing Bromopol (milk preservative; D&F Control Systems, San Ramon, CA, USA) was stored at 4°C for later analysis of milk composition. Blood samples were obtained at 09:00 h on 28 and 4 days prior to calving, at parturition, and 14 and 28 days after calving into evacuated 10-ml test tubes (Vacutainer, Becton Dickinson, Rutherford, NJ, USA) containing or without ethylene diamine tetraacetic acid (EDTA) by venipuncture of the coccygeal vessels, respectively. Blood samples were stored at room temperature for less than 3 h prior to the test. EDTA blood samples were used for the determination of immune cell counts. The tubes without EDTA were centrifuged at 3,000 × g for 12 min in a refrigerated centrifuge at 4°C. Serum was separated and transferred to 2-ml plastic scintillation vials and stored at –80°C for further analysis.

### Chemical Analysis

The analytical DM content of the TMR was determined by oven-drying at 135°C for 2 h (16). The crude protein contents were determined using an Elementar Rapid N Exceed (Elementar, Germany) according to the manufacturer’s instructions (17). The TMR samples were also analyzed for acid detergent fiber (ADF) (16) and neutral detergent fiber (NDF) (18) using the ANKOM 2000i automatic fiber analyzer (Beijing Anke Borui Technology Co. Ltd., Beijing, China). All chemical analyses were performed in duplicate.

Colostrum and milk samples were collected and analyzed for concentrations of crude protein (CP) by infrared spectroscopy (Foss Electric, Hillerod, Denmark). Calcium (Ca) and zinc (Zn) contents were determined by inductively coupled plasma–optical emission spectrometry (iCAP6300, Thermo Fisher company, USA) according to the methods described by Melton et al. (19). The colostrum and milk immunoglobulin (IgG, IgA, IgM) lactoperoxidase and lysozyme were measured using an enzyme-labeled instrument (BioTeck, VT, USA) for ELISA analysis with respective bovine ELISA kits (WSJH40101A, Beijing Laibotairui Technology Co. Ltd.) according to the instruction methods. The coefficients of variation of inter-assay and intra-assay in ELISA kits were 5.1% and 4.0%, respectively. The lactoferrin quantification in colostrum or milk was determined by high-performance liquid chromatography with fluorescence detection (U300, DIONEX company, USA) according to a simple immunoaffinity magnetic purification method described in Pang et al. (20).

The serum concentrations of interleukin (IL)-1β, IL-2, IL-6, IL-10, and tumor necrosis factor (TNF)-α were measured using an enzyme-labeled instrument (BioTeck, USA) with respective ELISA kits (WSJH40014A, Beijing Laibotairui Technology Co. Ltd.) according to the instruction methods. The coefficients of variation of inter-assay and intra-assay in ELISA kits were 4.8% and 4.2%, respectively.

For the determination of differential immune cell counts, 200 ml of blood were incubated with 5 ml of erythrocyte-lysing solution (8.26 g NH_4_Cl, 1.09 g NaHCO_3_, 0.037 g Na_2_ EDTA, and 1,000 ml A. dest.) for 5 min (room temperature). Then, the cells were pelleted by centrifugation (200 × g, 5 min) and resuspended in 1.2 ml of PBS (10 g NaCl, 0.25 g KCl, 0.25 g KH_2_PO_4_, 1.8 g Na_2_ HPO_4_ * 2H_2_O, and 1,000 ml A. dest.). Analysis was performed on a blood analyzer (BC-2800vet, Mindray company, China). Electronic gates were set according to the light scatter characteristics of lymphocytes, monocytes, and neutrophil granulocytes, and the proportion of the respective cell type was read.

### Statistical Analysis

The normal distribution of the data was checked using Proc UNI-VARIATE (release 9.1, SAS Institute Inc.). Commercial milk data were obtained from 14 to 28 days of milking. Data of colostrum and milk were subjected to ANOVA using the MIXED procedure of SAS, relevant model as follows:


$${\text{Y}}_{\text{ijk}}=\text{μ}+{\text{α}}_{\text{i}}+{\text{β}}_{\text{j}}+{\text{τ}}_{k}+{\left(\text{αβ}\right)}_{\text{ij}}+e_{\text{ijk}},$$

where Y_ijk_ is the dependent variable, μ is the overall mean, αi is the treatment effect (i = 1, 2, 3), β_j_ is the effect of milk (j = colostrum and commercial milk), τ_k_ is the random effect of cow, (αβ)_ij_ is the interaction effect of treatment and milk, and e_ijk_ is the residual error.

Data of blood parameters were analyzed using the model as follows:


$${\text{Y}}_{\text{ijk}}=\text{μ}+{\text{α}}_{\text{i}}+{\text{β}}_{\text{j}}+{\text{τ}}_{k}+{\left(\text{αβ}\right)}_{\text{ij}}+e_{\text{ijk}},$$

where Y_ijk_ is the dependent variable, μ is the overall mean, αi is the treatment effect (i = 1, 2, 3), β_j_ is the effect of sampling time for prepartum maternal cows (−28, −4, at parturition) or postpartum maternal cows (at parturition, 14, 28) or neonatal calves (0 h, 24 h), τ_k_ is the random effect of cow, (αβ)_ij_ is the interaction effect of treatment and sampling time, and e_ijk_ is the residual error.

The repeated measurement option in time was used with cow nested within treatment as the repeated subject for variables with repeated measurements. Differences among treatments were tested for significance using Tukey’s honestly significant difference. The correlation analysis using Pearson’s correlation coefficient was calculated by SPSS Statistics (V26, IBM, USA). Effects were considered significant at *p* < 0.05, whereas a tendency was assumed for 0.05 ≤ *p* ≤ 0.10. Data are expressed as means and SEM.

## Results

### Milk or Colostrum Production and Composition

Colostrum means colostrum samples obtained at the first milking after calving; commercial milk means milk samples obtained at 14 days and 28 days post-calving. Compared with CON, dairy cows in HN3 and HN6 had significantly higher commercial milk yield (*p* < 0.01), while it was not the case for colostrum yield (**Table 1**). There were no differences between groups in the concentrations of lactoferrin, lysozyme, Zn, and Ca in colostrum and commercial milk. However, the peroxidase concentration in commercial milk was greater (*p* = 0.02) in the HN3 treatment than that in the CON treatment (**Table 1**). Furthermore, the content of protein, lactoferrin, and Ca in commercial milk was significantly lower (*p* < 0.01) than that in colostrum, while the peroxidase and lysozyme concentrations in commercial milk were higher (*p* < 0.01) than those in colostrum (**Table 1**). The overall effect of Ca in HN6 was higher (*p* < 0.05) than that in CON, but no difference was observed in colostrum and commercial milk. Interestingly, the overall effect of Zn in HN3 was higher (*p* < 0.05) than that in CON, but no difference was observed in colostrum and commercial milk.

**Table 1:** Effects of dietary n-6 or n-3 PUFA on milk composition of transition cows from 28 days before calving to 28 days postpartum.

Item^1^	Diet^2^			SEM^3^	*p*-value		
	CON	HN6	HN3		Diet^3^	Time^3^	INT^3^
Production (kg)							
Colostrum	4.5	5.17	6.14	0.43	0.32	NA^4^	NA
Commercial milk	40.97^b^	46.23^a^	47.06^a^	0.78	<0.01	NA	NA
Overall	27.29^b^	32.54^a^	32.54^b^	1.98	0.01	<0.01	0.12
Protein (mg/100 g)							
Colostrum	17.63	15.18	15.48	0.76	0.39	NA	NA
Commercial milk	3.34	3.39	3.21	0.06	0.32	NA	NA
Overall	8.53	7.32	7.30	1.06	0.14	<0.01	0.14
Lactoferrin (mg/100 g)							
Colostrum	166.50	201.70	132.95	35.74	0.77	NA	NA
Commercial milk	3.55	3.10	3.45	0.40	0.90	NA	NA
Overall	57.87	69.30	46.62	17.44	0.56	<0.01	0.56
Lysozyme (μg/ml)							
Colostrum	0.37	0.33	0.57	0.11	0.68	NA	NA
Commercial milk	2.84	3.57	2.88	0.30	0.56	NA	NA
Overall	2.01	2.49	2.11	0.30	0.82	<0.01	0.67
Peroxidase (μg/ml)							
Colostrum	0.47	0.46	1.24	0.22	0.28	NA	NA
Commercial milk	9.72^b^	10.66^b^	14.98^a^	0.83	0.02	NA	NA
Overall	8.01	7.26	9.03	1.04	0.60	<0.01	0.88
Zn (mg/kg)							
Colostrum	18.83	17.88	22.50	0.95	0.10	NA	NA
Commercial milk	4.09	4.58	4.39	0.15	0.44	NA	NA
Overall	9.00^b^	9.01^b^	10.43^a^	1.27	<0.01	<0.01	<0.01
Ca (g/kg)							
Colostrum	2.21	2.47	2.37	0.06	0.19	NA	NA
Commercial milk	1.23	1.33	1.28	0.02	0.26	NA	NA
Overall	1.55^b^	1.71^a^	1.65^ab^	0.09	0.02	<0.01	0.43

^1^ Colostrum means the first milking yield after calving; commercial milk means 14 days post-calving to 28 days post-calving. Zn, Zinc; Ca, Calcium.

^2^ CON, control treatment (saturated fatty acid source), n = 12; HN3, extruded flaxseed treatment (n-3 PUFA source), n = 12; HN6, extruded soybean treatment (n-6 PUFA source), n = 13.

^3^ SEM, standard error of the mean; Diet, diet treatment effect; Time, the colostrum or commercial milk effect; INT, the interaction effect between diet effect and milk effect. Values in the same row denoted by different superscript lowercase letters indicate significant differences (p < 0.05) between treatments, whereas those denoted by the same letters or no letters are not significantly different (p > 0.05).

^4^ NA, not applicable.

### Blood Immune Cell Counts

**Figure 1:**
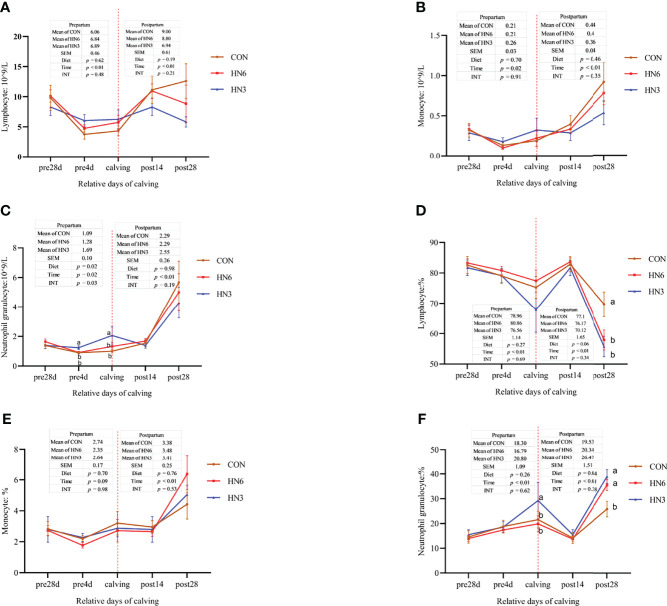
Blood immune cell counts in maternal cows. CON: control treatment (saturated fatty acid source), n = 12. HN3: extruded flaxseed treatment (n-3 PUFA source), n = 12. HN6: extruded soybean treatment (n-6 PUFA source), n = 13. SEM, standard error of the mean; Diet, diet treatment effect; Time, the calving time effect; INT, the interaction effect between diet effect and time effect. Values in the same sampling time point denoted by different superscript lowercase letters indicate significant differences (*p* < 0.05) between treatments, whereas those denoted by the same letters or no letters are not significantly different (*p* > 0.05). **(A–C)**: the blood immune cell counts in maternal cows; **(D–F)**: the percentage of blood immune cell in maternal cows.

During the prepartum period, the lymphocyte (*p* < 0.01) and monocyte (*p* = 0.02) were decreased in the three treatments over time (**Figure 1**). The neutrophilic granulocyte cell counts also decreased (*p* = 0.02) in CON and HN6 during the prepartum period but increased (*p* = 0.02) in HN3 at parturition (**Figures 1A–C**). The lymphocyte, monocyte, and neutrophilic granulocyte cell counts began to increase (*p* < 0.01) in the three treatments from calving to 28 days postpartum (**Figures 1A–C**). There were no treatment effects on total cell counts of lymphocytes and monocytes during the prepartum and postpartum periods, while the neutrophilic granulocyte cell counts were higher in HN3 than those in the other two treatments in pre-calving cows (**Figures 1A–C**). The lymphocyte percentage was decreased and neutrophilic granulocyte percentage was increased (*p* < 0.01) in each treatment from 28 days pre-calving to calving day. No differences were observed in the three diets for lymphocyte, monocyte, and neutrophilic granulocyte cell percentage before calving (**Figures 1D–F**). The lymphocyte percentage was highest at 14 days postpartum and lowest at 28 days postpartum, whereas the neutrophilic granulocyte percentage was lowest at 14 days postpartum and highest at 28 days postpartum (**Figures 1D–F**). The HN3 group tended to have a lower level (*p* = 0.06) of the lymphocyte percentage and a higher level (*p* = 0.04) of the neutrophilic granulocyte percentage during the postpartum period when compared with those in CON cows (**Figures 1D–F**). Especially, the lymphocyte percentage in HN3 was lower (*p* < 0.05) and the neutrophilic granulocyte percentage (*p* < 0.05) was higher at 28 days postpartum than those in CON, but no difference occurred between treatments at 14 days postpartum.

**Figure 2:**
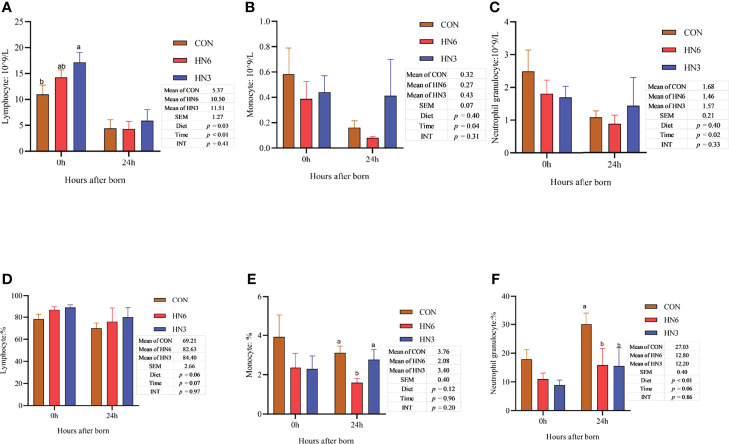
Blood immune cell counts in neonatal calves. CON: control treatment (saturated fatty acid source), n = 12. HN3: extruded flaxseed treatment (n-3 PUFA source), n = 12. HN6: extruded soybean treatment (n-6 PUFA source), n = 13. SEM, standard error of the mean; Diet, diet treatment effect; Time, the calving time effect; INT, the interaction effect between diet effect and time effect. Values in the same sampling time point between treatments denoted by different superscript lowercase letters indicate significant differences (*p* < 0.05), whereas those denoted by the same letters or no letters are not significantly different (*p* > 0.05). **(A–C)**: the blood immune cell counts in neonatal calves; **(D–F)**: the percentage of the blood immune cell in neonatal calves.

The monocyte and neutrophilic granulocyte cell counts in neonatal calves were not affected by diet treatments, while the lymphocyte cell counts in blood were greater (*p* < 0.05) in HN3 than those in CON in neonatal calves (**Figures 1A–C**). All of the immune cell counts in neonatal calves decreased (*p* < 0.05) at 24 h post-calving (**Figures 2A–C**). The HN6 and HN3 tended to increase (*p* = 0.06) the lymphocyte cell percentage and reduce (*p* < 0.01) the neutrophilic granulocyte cell percentage in neonatal calves when compared with CON treatment. The neutrophilic granulocyte cell percentage in HN6 and HN3 in neonatal calves after 24 h from birth was lower (*p* < 0.05) than that in CON, whereas there was no difference at 0 h. The HN6 group decreased (*p* < 0.05) the monocyte cell percentage in neonatal calves after 24 h from birth, but no difference was found at 0 h. The lymphocyte cell percentage did not differ among the three groups. There was a tendency to a lower (*p* = 0.06) percentage of lymphocyte cell percentage and a higher (*p* = 0.06) neutrophilic granulocyte cell percentage in calves for each treatment at 24 h after calving (**Figures 2D–F**).

### Blood Immunoglobulin

**Figure 3:**
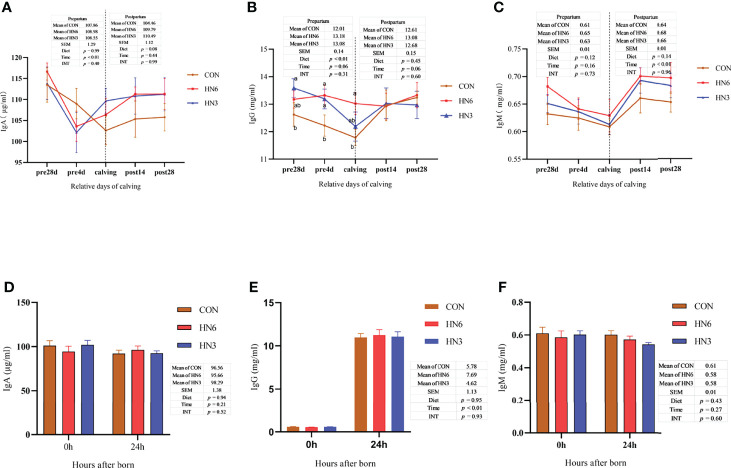
Blood immunoglobulin in maternal and neonatal cows. CON: control treatment (saturated fatty acid source), n = 12. HN3: extruded flaxseed treatment (n-3 PUFA source), n = 12. HN6: extruded soybean treatment (n-6 PUFA source), n = 13. SEM, standard error of the means; Diet, diet treatment effect; Time, the calving time effect; INT, the interaction effect between diet effect and time effect. IgA, immunoglobulin A; IgG, immunoglobulin G; IgM, immunoglobulin M. **(A–C)** The blood immunoglobulin in maternal cows; **(D–F)** the blood immunoglobulin in neonatal calves. Values in the same sampling time point between treatments denoted by different superscript lowercase letters indicate significant differences (*p* < 0.05), whereas those denoted by the same letters or no letters are not significantly different (*p* > 0.05).

The IgG concentration in the serum of maternal cows was significantly greater (*p* < 0.01) in HN6 at pre4d and calving than that in CON, whereas the IgA and IgM concentrations were not altered by the diet treatments during the whole transition period (**Figures 3A–C**). There were no significant differences in IgG and IgM in serum between groups during postpartum, while IgA tended to increase (*p* = 0.08) in HN3 and HN6 when compared with that in CON maternal cows (**Figures 3A–C**). The IgA in each treatment maternal cow significantly declined (*p* < 0.01) from 28 days pre-calving to parturition but was not changed from calving to 28 days postpartum (**Figure 3A**). The serum IgG was decreased (*p* = 0.06) and increased (*p* = 0.06) in a tendency in maternal cows during prepartum and postpartum, respectively (**Figure 3B**). The IgM in serum in maternal cows did not differ significantly from 28 days pre-calving to calving but had a significant increase (*p* < 0.01) from calving to 28 days postpartum (**Figure 3C**).

**Figure 4:**
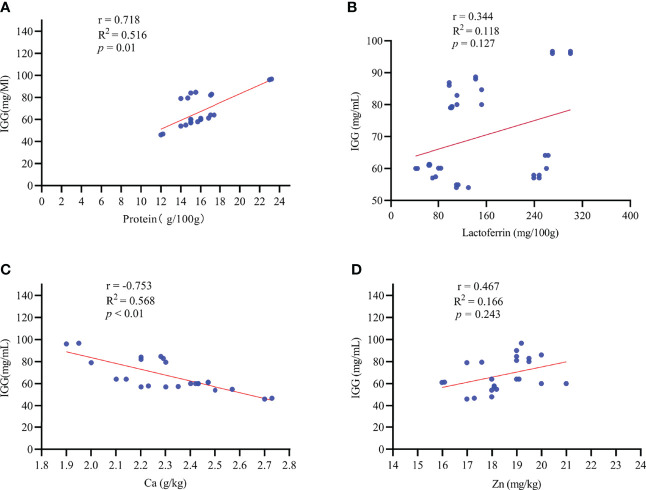
The correlation between colostrum IgG content and colostrum composition. r: Pearson correlation coefficient. Effects were considered significant at *p* < 0.05, whereas a tendency was assumed for 0.05 ≤ *p* ≤ 0.10. n = 35. IGG, immunoglobulin G; Ca, calcium; Zn, Zinc. **(A–D)**: the correlation of macro-element and trace element with IgG concentration in colostrum.

There were no observed differences between groups in concentrations of serum IgA, IgG, and IgM in neonatal calves (**Figures 3D–F**). The post-calving time and colostrum feeding did not affect the IgA and IgM in calf serum (**Figures 3D–F**). As expected, the IgG concentration was increased in neonatal calf serum at 24 h after calving (**Figure 4E**).

### Milk Immunoglobulin

The IgA and IgM concentrations in colostrum and commercial milk were not shifted by diet treatments, while the IgA and IgM concentrations in commercial milk were greater (*p* < 0.01) than those in colostrum (**Table 2**). The IgG in colostrum was significantly lower (*p* < 0.01) in HN6 and HN3 than that in CON treatment (**Table 2**). Meanwhile, the IgG concentrations in colostrum were significantly higher (*p* < 0.01) than those in commercial milk (**Table 2**). However, the IgG yield in colostrum or commercial milk was not significantly affected by diet treatments (**Table 2**). Moreover, the average IgG yield obtained in colostrum and commercial milk was higher (*p* = 0.04) in HN3 than that in HN6 (**Table 2**).

**Table 2:** Effects of dietary n-6 or n-3 PUFA on milk immunoglobulin of transition cows from 28 days before calving to 28 days postpartum.

Item^1^	Diet^2^			SEM^3^	*p*-value		
	CON	HN6	HN3		Diet^3^	Time^3^	INT^3^
IgA (μg/ml)							
Colostrum	0.17	0.21	0.35	0.04	0.18	NA^4^	NA
Commercial milk	0.63	0.70	0.72	0.05	0.73	NA	NA
Overall	0.48	0.54	0.60	0.05	0.35	<0.01	0.78
IgG (mg/ml)							
Colostrum	85.90^a^	56.50^b^	59.15^b^	4.32	<0.01	NA	NA
Commercial milk	0.94	1.06	0.97	0.05	0.64	NA	NA
Overall	29.26^a^	19.54^b^	20.37^b^	5.46	<0.01	<0.01	<0.01
IgM (mg/ml)							
Colostrum	0.13	0.18	0.24	0.03	0.43	NA	NA
Commercial milk	0.46	0.63	0.55	0.07	0.63	NA	NA
Overall	0.35	0.48	0.44	0.06	0.64	<0.01	0.85
IgG (g)							
Colostrum	301.28	236.81	412.13	42.87	0.26	NA	NA
Commercial milk	35.54	46.47	45.39	2.56	0.16	NA	NA
Overall	124.12^ab^	109.92^b^	167.64^a^	25.94	0.04	<0.01	0.05

^1^ Colostrum means colostrum samples obtained at the first milking after calving; commercial milk means milk samples obtained at 14 days and 28 days post-calving. IgA, immunoglobulin A; IgG, immunoglobulin G; IgM, immunoglobulin M.

^2^ CON: control treatment (saturated fatty acid source), n = 12; HN3: extruded flaxseed treatment (n-3 PUFA source), n = 12; HN6: extruded soybean treatment (n-6 PUFA source), n = 13.

^3^ SEM, standard error of the mean; Diet, diet treatment effect; Time, the colostrum or commercial milk effect; INT, the interaction effect between diet effect and milk effect. Values in the same row denoted by different superscript lowercase letters indicate significant differences (p < 0.05) between treatments, whereas those denoted by the same letters or no letters are not significantly different (p > 0.05).

^4^ NA, not applicable.

In addition, we found that the IgG concentration in colostrum was positively associated (r = 0.718, *p* = 0.01) with the protein concentration in colostrum, while it had a negative relation (r = -0.753, *p* < 0.01) with the Ca concentration in colostrum (**Figures 4A–C**). There was no significant correlation between the IgG concentration and the lactoferrin (r = 0.344, *p* = 0.127) or Zn (r = 0.467, *p* = 0.243) concentration in colostrum (**Figures 4B–D**).

### Blood Inflammatory Cytokines

**Figure 5:**
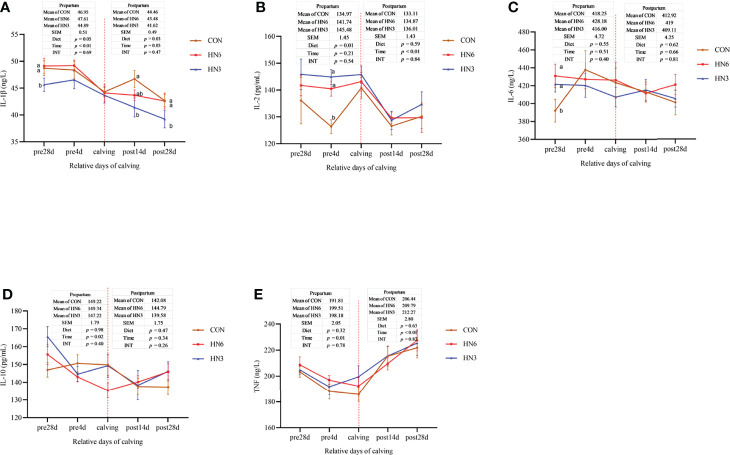
Blood inflammatory cytokines in maternal cows. CON: control treatment (saturated fatty acid source), n = 12. HN3: extruded flaxseed treatment (n-3 PUFA source), n = 12. HN6: extruded soybean treatment (n-6 PUFA source), n = 13. SEM, standard error of the mean; Diet, diet treatment effect; Time, the calving time effect; INT, the interaction effect between diet effect and time effect. IL, interleukin; TNF, tumor necrosis factor. **(A–E)**: the different blood inflammatory cytokines in maternal cows. Values in the same sampling time point between treatments denoted by different superscript lowercase letters indicate significant differences (*p* < 0.05), whereas those denoted by the same letters or no letters are not significantly different (*p* > 0.05).

The concentration of IL-1β in the serum of maternal cows in HN3 was lower during prepartum (*p* = 0.05) and postpartum (*p* = 0.03) compared with the ones in CON and HN6 (**Figure 5A**). The serum concentration of IL-2 in HN3 was greater (*p* = 0.01) than that in CON treatment in pre-calving cows, but there was no change in post-calving cows (**Figure 5B**). Dietary treatments did not affect the serum concentration of IL-6, IL-10, and TNF-α in prepartum or postpartum maternal cows (**Figures 5C–E**). An effect of time was observed for almost all variables in the prepartum or postpartum periods (**Figure 5**). The serum concentration of IL-1β in maternal cows was decreased during prepartum (*p* < 0.01) and the postpartum period (*p* = 0.05), IL-2 was decreased from parturition to 28 days postpartum, IL-10 was decreased from 28 days pre-calving to calving, and TNF was decreased from 28 days before calving to calving and then increased from calving until 28 days post-calving. There was no significant interaction between time and diet treatment for these inflammatory cytokines (**Figure 5**).

**Figure 6:**
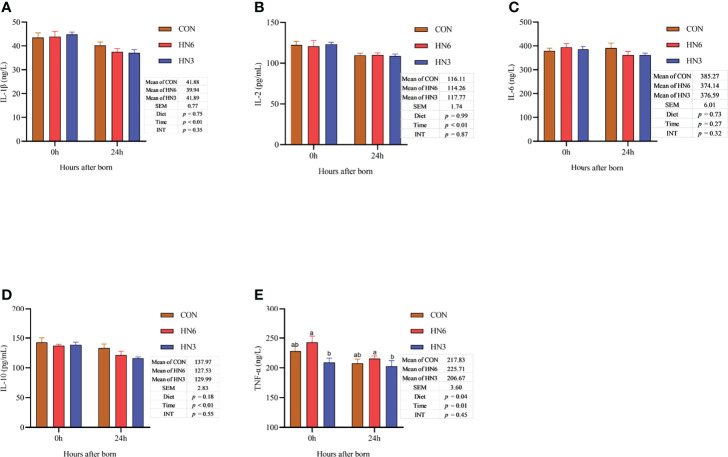
Blood inflammatory cytokines in neonatal calves. CON: control treatment (saturated fatty acid source), n = 12. HN3: extruded flaxseed treatment (n-3 PUFA source), n = 12. HN6: extruded soybean treatment (n-6 PUFA source), n = 13. SEM, standard error of the mean; Diet, diet treatment effect; Time, the calving time effect; INT, the interaction effect between diet effect and time effect. IL, interleukin; TNF-α, tumor necrosis factor α. **(A–E)**: the different blood inflammatory cytokines in neonatal calves. Values in the same sampling time point between treatments denoted by different superscript lowercase letters indicate significant differences (*p* < 0.05), whereas those denoted by the same letters or no letters are not significantly different (*p* > 0.05).

There was no difference in the neonatal calf serum concentration of inflammatory cytokines between diet treatments, except for TNF, which was decreased (*p* = 0.04) in HN3 compared with that in the HN6 treatment (**Figure 6**). After colostrum feeding, the concentration of the inflammatory cytokines in neonatal calves decreased (*p* < 0.05) at 24 h after calving, but the IL-6 was not altered (*p* = 0.27) by the calving time (**Figure 6**). No interactions between diet and calving time were observed for inflammatory cytokines (**Figure 6**).

## Discussion

In this part, we discussed about the effect of n-3 and n-6 PUFA on the IgG concentration and yield in colostrum. Moreover, we evaluated the influence of n-3 or n-6 PUFA addition on the immune cell counts in blood and inflammotary cytokines in serum in maternal cows and neonatal cows.

The protein percentage in colostrum and commercial milk did not differ between the dietary groups in our study and may be due to the protein level being similar in the diet between groups (**Table S1**). It also could be explained by a meta-analysis literature that concluded that protein was adequate in the diet to support the level of performance, and most studies showed no effect of oil supplementation on milk protein (21). In the present study, cows fed any source of PUFA (n-6 or n-3) compared with saturated FA-supplemented cows produced colostrum with lower IgG content, without affecting yield and chemical composition. Garcia et al. (22) reported that multiparous cows fed fat (saturated or rich in n-6 FA) produced colostrum with higher IgG concentration compared with parous cows not supplemented with fat. On the other hand, others reported that cows fed n-6 but not n-3 produced colostrum with higher IgG content compared with non-supplemented cows (23). Our findings and those of others were not in complete agreement on which fat enriched in n-6 PUFA had higher contents of IgG in colostrum than saturated FA. Moreover, the IgG content data (CON, 79.4–96.7 g/L vs. HN6, 46.8–61.3 g/L vs. HN3 54.9–64.1 g/L) obtained in our study were less than previous study values (fat-supplemented 126 g/L vs. non-fat-supplemented 98 g/L) (24). However, in the current study, the colostrum IgG production was not affected by the type of FA (CON, 169–386 g; HN6, 183.91–289.38 g; HN3, 224.49–494.45 g; *p* = 0.26). It meant that the PUFA treatment did not reduce the system to produce IgG yield. On the other hand, the protein concentration value in n-6 (15.18 g/100 g) or n-3 PUFA (15.48 g/100 g) treatment was at least 2 g/100 g lower (*p* = 0.32) than that of saturated FA (17.63 g/100 g), which was in line with our correlation analysis, and another study showed that the IgG content was highly positively related to protein in colostrum (25).

Our results also found that the IgG content was highly negatively correlated (r = -0.753, *p* < 0.01) with Ca content in colostrum. Zn plays an important role in enzyme systems, protein synthesis, and many other biochemical reactions (26). Even though the treatment did not shift the Zn concentration in colostrum or commercial milk, the HN3 significantly increased the overall Zn concentration. More Zn in milk fed to calves would benefit the growth of calves (27). It suggested that the diet added with HN3 could potentially increase the milk value for calves. As expected, the commercial milk content of protein, lactoferrin, Ca, and Zn was significantly lower than that of colostrum. The results were in line with the study of Georgiev (28), who demonstrated that the chemical composition of colostrum (solid nonfat extract, milk protein) decreases rapidly with time, so that by the third day postpartum, it is already similar to normal milk. Cow colostrum also contains components with a concentration lower than 1 mg/ml (trace components, i.e., enzymes, IgA, IgM, etc.). Despite their low concentrations, trace components are physiologically important for both the local protection of the udder and the growth and development of neonates (28). In our study, the contents of lysozyme, peroxidase, IgA, and IgM were higher in commercial milk than those in colostrum. It could be explained by Levieux (29), who revealed that trace elements decrease abruptly during the first milking postpartum and then increase in late lactation that may be due to the udder involution before calving. In addition, it also has been confirmed that lactoperoxidase content in bovine colostrum is low and reaches its maximum concentration between 3 and 5 days postpartum (30). Moreover, the diet enriched in n-3 PUFA increased the commercial milk lactoperoxidase content. Lactoperoxidase is responsible for catalyzing the oxidation of specific molecules using H_2_O_2_ to generate reactive products with high antibacterial activity (31). It indicated that the colostrum was vulnerable to bacterial contamination, while n-3 PUFA supplementation could increase the bacteriostatic ability of commercial milk.

In this study, the commercial milk production was increased in cows fed n-3 or n-6 sources of PUFA when compared with those fed saturated FA sources. It was supported by a previous study that showed that supplementation after calving with high n-3 PUFA oilseed increased milk production of dairy cows in the early stage of lactation (32). Furthermore, Petit (33) also found that the milk production from the cows fed n-3 PUFA source oilseed for the first 16 weeks of lactation was similar to the milk yield of cows fed n-6 PUFA source oilseed but higher than that of those fed protected palm oil. However, our results were not completely in line with a recent meta-analysis literature that suggested that the effects of n-3 PUFA source supplementation in the diet on milk production of dairy cows seem to be neutral (34). These different results could be explained by Onetti and Grummer (35), who found that there was an interaction between the stage of lactation and the amount of supplemental fat observed, since supplemental fat increases milk production of dairy cows in the early stage of lactation but not that of cows in the mid-stage lactation, where milk fat depression occurred. In addition, we consider that the n-3 or n-6 PUFA could improve the colostrum and milk production that may be partially due to the PUFA addition improving the mammary gland recovery when cows transferred from pre-calving to post-calving. It was supported by Hilakivi–Clarke et al. (36) who reported that the PUFA could benefit the development of mammary glands.

Impaired neutrophil, mononuclear leukocytes, and lymphocyte activity were observed in cows during the periparturient period (37, 38). Mononuclear leukocytes protect the body against invading pathogens and play an important role in innate immunity. In contrast, lymphocytes play a critical role in cell- and antibody-mediated adaptive immune responses (39). The consequences of impaired function and killing activity of neutrophils on disease incidence also have been reviewed elsewhere (40–42), which suggested that neutrophils could modulate the inflammatory microenvironment and exert direct antimicrobial action. In this study, we found that the immune cell counts (i.e., mononuclear leukocytes, lymphocytes, and neutrophils) in maternal cows were reduced during the prepartum period compared with those in the postpartum period, which was in line with the reports of Harp et al. (43), Kimura et al. (44), Nagahata et al. (45), and Roche et al. (46). Our findings and these studies suggested that the function and killing activity of immune cells may be reduced by decreasing the numbers of the immune cells as a function of reduced proliferation during the transition period. Interestingly, the diet supplemented with high n-3 PUFA increased the maternal cows’ neutrophil counts during prepartum and calving and elevated neutrophil percentage at calving and during postpartum. It suggested that the n-3 PUFA could potentially improve the body’s defense against pathogen invasion by increasing the immune cell counts in maternal cows during the transition period.

Fetal calves are predominantly protected by the innate immune system. The innate immune response mediated by phagocytic cells (neutrophils and macrophages) does not fully develop until late gestation, and there is a decline in functional capacity as gestation approaches because of the increase in fetal cortisol levels (47). The number of peripheral blood T cells dramatically decreases beginning 1 month before calving as they traffic and populate lymphoid tissues of fetal calves (48). A subsequent decrease in the number of immune cells from calving to 24 h after calving (post colostrum feeding) in newborn calves was found in our study. It could be explained by Chase et al. (49), who found that the circulating humoral components of the innate system in newborn calves were quickly diminished to less than 20% of the level circulating in adult cows at 1 day of age. And then, the levels of complement in circulation gradually increase and, by 1 month of age, increase to approximately 50% of the level in adults (50). Therefore, the newborn calves were very susceptible to infection due to the inhibitory innate immune function. It is crucial to feed newborn calves with sufficient colostrum (enriched in IgG) as soon as possible. The data observed in this study showed that the diet enriched in n-3 PUFA increased the lymphocyte cell counts in neonatal calves. It suggested that the n-3 PUFA could potentially increase the neonatal calves’ lymphocyte immune function.

Inflammation is an evolutionarily conserved response underlying many physiological and pathological processes. In response to the stimuli associated with infection and tissue injury, components of the innate and adaptive immunity initiate coordinated responses and trigger inflammation (51). However, an exaggerated inflammatory response would result in negative effects on the transition cow. The previous study showed that all cows experience some degree of systemic inflammation associated with the pro-inflammatory cytokine increase several days after parturition, and the magnitude and potential persistence of the inflammatory state vary widely among cows, and several studies have linked the degree of postpartum inflammation to increased disease risk and decreased whole-lactation milk production (52). These results were not completely consistent with those of our study; we did not find a dramatic increase in pro-inflammatory cytokines IL-1β and IL-6. In addition, the anti-inflammatory cytokines IL-2 and IL-10 were observed to decrease after calving, which indicated that calving can decrease the ability to control the inflammation status. Interestingly, we found that the n-3 PUFA treatment could decrease the IL-1β concentration during postpartum and increase IL-2 during prepartum. These results agreed with those of Zhao et al. (53), who reported that the concentrations of n-3 PUFA capable of binding Nuclear factor kappa B (NFκB) declined during the transition period and provided Nuclear factor kappa B (NFκB) effects such as limiting the production of inflammatory cytokines such as IL-1β, IL-6, and TNF. It might also explain why n-3 PUFA treatment decreased the blood TNF in newborn calves, which could benefit the subsequent health and growth of calves. Fortunately, we found that the n-3 PUFA could reduce the inflammation reaction of maternal cows during the transition period by decreasing IL-1β and increasing IL-2, which would help the dairy cows to transfer smoothly from pregnancy to lactation and partially explained the increase of milk production in n-3 PUFA treatment.

## Conclusion

Our results suggested that the diets with similar levels of fat, fiber, and protein, but containing extruded flaxseed (n-3 PUFA source) or extruded soybean (n-6 PUFA source) instead of hydrogenated saturated FA (enriched in C16:0 saturated FA), increased the milk production of cows without altering the protein content and decreased the IgG content without altering the IgG yield in colostrum. The diet with the n-3 PUFA source could potentially increase the body’s ability to defend against pathogen invasion in maternal cows by increasing the neutrophil numbers and percentage during the transition period. Meanwhile, the diet with n-3 PUFA source could potentially reduce the inflammation of the maternal cows during the postpartum period and decrease the neonatal calves’ inflammatory cytokine TNF content in serum. It also suggested that the highest milk production in n-3 PUFA treatment may partially be due to these beneficial alterations.

## Data Availability Statement

The original contributions presented in the study are included in the article/**Supplementary Material**. Further inquiries can be directed to the corresponding authors.

## Ethics Statement

The animal study was reviewed and approved by The China Agricultural University Laboratory Animal Welfare and Animal Experimental Ethical Inspection Committee.

## Author Contributions

Conceptualization, SL and WW; methodology, YH, and ZC; software, XS; validation, SL; formal analysis, XS; investigation, XS, ZY, YZ, QW, ZW, and CG; resources, CG; data curation, XS; writing—original draft preparation, XS; writing—review and editing, XS, YH and YW; visualization, XS; supervision, SL; project administration, SL; and funding acquisition, SL. All authors have read and agreed to the published version of the manuscript.

## Funding

This research was funded by National Natural Science Foundation of China (32130100), and China Agriculture Research System of MOF and MARA.

## Conflict of Interest

The authors declare that the research was conducted in the absence of any commercial or financial relationships that could be construed as a potential conflict of interest.

## Publisher’s Note

All claims expressed in this article are solely those of the authors and do not necessarily represent those of their affiliated organizations, or those of the publisher, the editors and the reviewers. Any product that may be evaluated in this article, or claim that may be made by its manufacturer, is not guaranteed or endorsed by the publisher.
