# Supplementary material for: The Blood Immune Cell Count, Immunoglobulin, Inflammatory Factor, and Milk Trace Element in Transition Cows and Calves Were Altered by Increasing the Dietary n-3 or n-6 Polyunsaturated Fatty Acid Levels
Source: Front Immunol. 2022 Jul 7;13:897660. doi: 10.3389/fimmu.2022.897660 (PMC9300944; doi:10.3389/fimmu.2022.897660)
Supplement: Supplementary file 1 [file Table_1.docx]

**Table S1.** The ingredients and chemical composition of the close-up and milking cow TMR (DM basis)

| Item | Prepartum | | |  | Postpartum | | | | |
| --- | --- | --- | --- | --- | --- | --- | --- | --- | --- |
|  | CON | HN6 | HN3 |  | CON |  | HN6 |  | HN3 |
| Ingredients (%) |  |  |  |  |  | |  | |  |
| Oat hay | 47.82 | 47.62 | 47.62 |  |  | |  | |  |
| Alfalfa hay |  |  |  |  | 10.35 | | 10.24 | | 10.24 |
| Corn silage | 21.70 | 21.55 | 21.61 |  | 30.43 | | 30.06 | | 30.10 |
| Alfalfa silage |  |  |  |  | 7.84 | | 7.71 | | 7.76 |
| Corn fine | 3.88 | 2.99 | 2.74 |  | 9.46 | | 10.35 | | 9.37 |
| Soybean Hulls | 7.72 | 7.69 | 7.70 |  | 2.65 | | 2.61 | | 2.63 |
| Corn steam flakes |  |  |  |  | 10.32 | | 10.15 | | 10.21 |
| Soybean meal | 7.61 | 1.88 | 6.58 |  | 16.15 | | 9.23 | | 14.48 |
| Corn gluten feed | 4.08 | 4.07 | 4.06 |  |  | |  | |  |
| Urea | 0.61 | 0.61 | 0.61 |  |  | |  | |  |
| Cottonseed fuzzy |  |  |  |  | 5.36 | | 5.30 | | 5.33 |
| Sugarcane molasses |  |  |  |  | 2.59 | | 2.55 | | 2.56 |
| Diamond V XP | 0.26 | 0.26 | 0.26 |  | 0.16 | | 0.16 | | 0.16 |
| Hydrogenated FA | 1.00 |  |  |  | 1.00 | |  | |  |
| Extruded soybean |  | 8.00 |  |  |  | | 8.00 | |  |
| Extrude flaxseed |  |  | 3.50 |  |  | |  | | 3.50 |
| Dry cow premix | 4.31 | 4.32 | 4.31 |  |  | |  | |  |
| DCAD supplement | 1.01 | 1.01 | 1.01 |  |  | |  | |  |
| Milking cow premix |  |  |  |  | 3.69 | | 3.64 | | 3.66 |
| Nutrition level |  |  |  |  |  | |  | |  |
| DM (LeBlanc et al.) | 12.97 | 13.02 | 13.02 |  | 17.27 | | 17.41 | | 17.26 |
| CP(%) | 15.56 | 15.47 | 15.52 |  | 17.38 | | 17.31 | | 17.39 |
| NE_L_(Mcal/kg) | 1.27 | 1.28 | 1.28 |  | 1.68 | | 1.69 | | 1.71 |
| NFC(%) | 30.36 | 29.46 | 29.30 |  | 41.77 | | 41.53 | | 41.35 |
| NDF(%) | 49.07 | 49.82 | 49.67 |  | 30.39 | | 32.65 | | 31.64 |
| EE(%) | 3.70 | 3.72 | 3.82 |  | 5.43 | | 5.35 | | 5.49 |
| C16:0 (g/d) | 91.13 | 46.89 | 43.36 |  | 196.21 | | 98.60 | | 94.02 |
| n-6(g/d) | 83.53 | 156.65 | 87.91 |  | 191.60 | | 347.64 | | 226.57 |
| n-3(g/d) | 49.63 | 51.21 | 136.52 |  | 39.33 | | 42.57 | | 142.56 |
| n-6: n-3 | 1.68: 1 | 3.05: 1 | 0.64: 1 |  | 4.87: 1 | | 8.16: 1 | | 1.59: 1 |

DM: Dry matter, CP: Crude protein, NE_L_: Net energy of lactation, a calculated value according to NRC (2001), NFC: Non-fibrous carbohydrate, NDF: Neutral detergent fiber, EE: Ether extract, UFA: Unsaturated fat acid, n-6: C18:2n6 (Linoleic acid, LA), n-3: C18:3n3 (α- Linolenic acid, ALA). Dry cow premix: 1 kg of premix included vitamin A 918,750 IU, vitamin D3 253,125 IU, vitamin E 8,066 mg, niacin 6,650 mg, Ca 35 g, P 20 g, Cu 1146mg, Mn 4248 mg, Zn 5362 mg, I 45 mg, Se 36 mg. Milking cow premix: 1 kg of premix included vitamin A 440,000 IU, vitamin D3 110,000 IU, vitamin E 4000 IU, niacin 400 mg, Ca 152 g, P 41 g, Cu 750 mg, Mn 1140 mg, Zn 2970 mg, I 30 mg, Se 24 mg. DCAD supplement: -107.43 (mEq/kg) = (Na%/0.023+K%/0.039-Cl%/0.0355-S%/0.016). CON: Control treatment, HN6: High n-6 polyunsaturated fatty acid (PUFA) treatment, HN3: High n-3 PUFA treatment.
