# Supplementary material for: The Blood Immune Cell Count, Immunoglobulin, Inflammatory Factor, and Milk Trace Element in Transition Cows and Calves Were Altered by Increasing the Dietary n-3 or n-6 Polyunsaturated Fatty Acid Levels
Source: Front Immunol. 2022 Jul 7;13:897660. doi: 10.3389/fimmu.2022.897660 (PMC9300944; doi:10.3389/fimmu.2022.897660)
Supplement: Supplementary file 2 [file Table_2.docx]

**Table S2.** The information on cows was not included in the statistical analysis

| Item | CON^3^ | HN6^4^ | HN3^5^ |  |
| --- | --- | --- | --- | --- |
| Calved early, cow^1^ | 0 | 1 | 1 |  |
| Mastitis, cow | 0 | 1 | 0 |  |
| DA^2^ | 1 | 0 | 1 |  |
| lameness | 1 | 0 | 1 |  |
| pneumonia | 1 | 0 | 0 |  |

^1^ Calved early: The cow calved early 15 days before the expecting calving date.

^2^ DA: Displacement of the abomasum.

^3^ CON: Control treatment.

^4^ HN6: High n-6 polyunsaturated fatty acid (PUFA) treatment.

^5^ HN3: High n-3 PUFA treatment.
