# Supplementary material for: The Blood Immune Cell Count, Immunoglobulin, Inflammatory Factor, and Milk Trace Element in Transition Cows and Calves Were Altered by Increasing the Dietary n-3 or n-6 Polyunsaturated Fatty Acid Levels
Source: Front Immunol. 2022 Jul 7;13:897660. doi: 10.3389/fimmu.2022.897660 (PMC9300944; doi:10.3389/fimmu.2022.897660)
Supplement: Supplementary file 3 [file Table_3.docx]

**Table S3.** The information of the cows at beginning (n=37)

| Item | CON^1^ | HN6^2^ | HN3^3^ | p-value | Overall |
| --- | --- | --- | --- | --- | --- |
| Parity(± SD) | 2.69±0.89 | 2.29±0.69 | 2.51±0.48 | *P* = 0.56 | 2.47±1.06 |
| Body weight (kg ± SD) | 754.53±55.21 | 756.23±45.62 | 765.43±56.55 | *P* = 0.78 | 759.23±65.89 |
| Body condition score(± SD) | 3.28±0.23 | 3.29±0.28 | 3.32±0.26 | *P* = 0.63 | 3.30±0.26 |
| Previous lacatation milk yield  (kg ± SD) | 10013.18±1460.39 | 9996.56±1064.84 | 10566.29±1551.00 | *P* = 0.70 | 10182.18±1664.90 |

^1^ CON: Control treatment.

^2^ HN6: High n-6 polyunsaturated fatty acid (PUFA) treatment.

^3^ HN3: High n-3 PUFA treatment.
