# Supplementary material for: The Blood Immune Cell Count, Immunoglobulin, Inflammatory Factor, and Milk Trace Element in Transition Cows and Calves Were Altered by Increasing the Dietary n-3 or n-6 Polyunsaturated Fatty Acid Levels
Source: Front Immunol. 2022 Jul 7;13:897660. doi: 10.3389/fimmu.2022.897660 (PMC9300944; doi:10.3389/fimmu.2022.897660)
Supplement: Supplementary file 4 [file Table_4.docx]

**Table S4.** Diarrhea condition was observed in calves within 14 days after calving

| Item | CON^1^ | HN6^2^ | HN3^3^ |  |
| --- | --- | --- | --- | --- |
| Diarrhea, calves | 4 | 4 | 5 |  |

^1^ CON: Control treatment.

^2^ HN6: High n-6 polyunsaturated fatty acid (PUFA) treatment.

^3^ HN3: High n-3 PUFA treatment.
